# A retrospective study of the prognostic value of MRI-derived residual tumors at the end of intensity-modulated radiotherapy in 358 patients with locally-advanced nasopharyngeal carcinoma

**DOI:** 10.1186/s13014-015-0401-0

**Published:** 2015-04-15

**Authors:** Yuxiang He, Qin Zhou, Lin Shen, Yajie Zhao, Mingjun Lei, Rui Wei, Liangfang Shen, Shousong Cao

**Affiliations:** Department of Oncology, Xiangya Hospital, Central South University, Hunan Province, No. 87, Xiangya Road, Changsha, Hunan Province 410008 P.R. China

**Keywords:** Nasopharyngeal carcinoma, Residual tumor, MRI, IMRT, Prognosis

## Abstract

**Objective:**

To retrospectively analyze the prognostic value of magnetic resonance imaging (MRI)-derived residual tumors after intensity-modulated radiation therapy (IMRT) in the patients with locally-advanced nasopharyngeal carcinoma.

**Methods:**

A total of 358 patients with locally-advanced nasopharyngeal carcinoma who received IMRT were classified as having residual tumors or no residual tumor based on MRI at the end of radiotherapy. The χ^2^ test, log-rank test, Cox proportional hazards regression model and Kaplan-Meir survival curves were used to investigate the relationship of clinicopathological features and residual tumors and to assess the prognostic value of residual tumors.

**Results:**

The 3-year overall survival (OS) rate was 73% in the residual tumor group and 90% in the no residual tumor group (HR 2.15, 95% CI 1.21-3.82,, *P* = 0.007); 3-year local relapse-free survival (LRFS) was 89% in the residual tumor group and 97% in the no residual tumor group (HR 4.46, 95% CI 1.61-12.38, *P* = 0.002); 3-year disease free survival (DFS) was 67% in the residual tumor group and 82% in the no residual tumor group (HR 2.21, 95% CI 1.40-3.48, *P* = 0.001). A high prescribed radiation dose (>73.92 Gy) did not increase the percentage volume of the GTVnx receiving 95% of the prescribed dose (GTVnx V95%) or improve any survival outcome.

**Conclusion:**

The presence of a residual tumor after IMRT was a significant negative independent prognostic factor for OS, LRFS and DFS. Although IMRT have improved the distribution of radiotherapy doses into the tumors, residual tumors detected by MRI after IMRT are still associated with poor prognosis in patients with advanced nasopharyngeal carcinoma.

## Background

The presence of residual tumors after treatment may provide important prognostic information [1] and may be associated with the radiosensitivity of the tumor. A prospective study of the correlation of regression rate and the probability of recurrence after radiation of neck node metastases in 47 patients by Bartelink showed that tumors with a slow regression rate had a high probability of recurrence [2]. Residual foci detected by clinical or imaging methods may be composed of cancer parenchyma-like cancer stem cells and delayed reproductive-dead cells, as well as other cell types including interstitial tissue-like fibrosis and mononuclear cells [3]. While reproductive-dead cells will disappear within several months, radio-resistant cancer stem cells are thought to be the root cause of relapse and metastasis [4], and the radio-response of the interstitial tissue is also associated with recurrence [3]. Given these possibilities, clinicians need to decide whether or not to increase the local dosage or provide timely additional adjuvant chemotherapy for patients with radiographically-visible residual tumors at the end of radiotherapy. Biopsy is the “gold standard” for diagnose of residual tumor. However, most the residual tumors are located outside the nasopharynx in the patients with locally-advanced nasopharyngeal carcinoma (NPC) so biopsy for the tumors is difficult to be obtained. Ng et al. [5] in a retrospectively reviewed study reported the MRI features of recurrent NPC in 72 patients who underwent MRI and showed a nasopharyngeal mass in 50 patients (69.4%) involved outside the nasopharynx including the parapharyngeal space (44.4%), nasal cavity (12.5%), paranasal sinuses (27.8%), oropharynx (4.2%), orbit (8.3%), infratemporal fossa (18.1%), skull base (59.8%), intracranial area (51.4%) and regional lymph nodes (15.3%). Moreover, the viable cells in residual tumors identified by pathology at the end of radiation may become died cells at later time and pathology cannot detect the depth of tumor invasion which is usually a poor indicator for prognosis [6]. Therefore, clinicians must rely on imaging methods to evaluate the therapeutic effect in most cases. Liauw et al. [7] examined the correlation between treatment response and neck dissection pathology, and reported that residual tumors had a negative predictive value of 77% for complete clinical response and 94% for radiographic complete response (rCR) in patients with head and neck cancer treated with radiotherapy. Magnetic resonance imaging (MRI) is the preferred modality for determining the extent of soft tissue, perineural infiltration, intracranial spread and skull base invasion of NPC [8]. It was reported that MRI had a higher accuracy for detecting residual and/or recurrent NPC at the primary tumor site than fluorodeoxyglueose positron emission tomography with computed tomography (FDG PET/CT) [9]. Therefore, it is worthy of investigating whether MRI-derived residual tumors are associated with the prognosis of patients with locally-advanced NPC to simplify and improve the diagnosis and treatment of NPC.

The purpose of this retrospective study of 358 patients with locally-advanced NPC was to compare the prognosis of patients with or without residual tumors based on MRI at the end of intensity-modulated radiotherapy (IMRT). The results may provide a basis for assessing the value of boost radiation or timely adjuvant chemotherapy for patients with residual tumors at the end of radiotherapy.

## Materials and methods

### Patients

A total of 358 patients with locally-advanced NPC (T3/T4N0-3M0) who received IMRT between August 2008 and December 2011 at Xiangya Hospital of Central South University (Changsha, Hunan province, China) were enrolled in this study. All patients were diagnosed by nasopharyngeal biopsy and nasopharyngeal and neck MRI examinations. In addition to CT/MRI examination of the nasopharynx and neck, the pre-treatment work-up also included a complete medical history, physical examination, chest X-ray and/or CT (all patients with N3 disease underwent a chest CT), B-ultrasound scan of the abdomen and neck, bone scan and routine laboratory analysis. The clinical characteristics of the patients are summarized in Table 1. In the study, 354 out of 358 cases were eligible for univariate and multivariate analyses due to 4 patients without MRI review after radiotherapy. Additionally, 346 cases were eligible for survival analysis due to 12 patients loss of follow-up. The study was approved by the ethics committee of Xiangya Hospital of Central South University (approval number 201111086).

**Table 1:** **Clinical characteristics of the patients and factors associated with residual tumors at the end of IMRT**

**Characteristic**	**Group**	**Patients without residual tumor (** ***n =*** **212)**	**Patients with residual tumor (** ***n =*** **142)**	***Χ*** ^***2***^	***P***
Age (y)	<50	138 (59.7%*)	93 (40.3%)	0.006	0.938
	≥50	74 (60.2%)	49 (39.8%)		
T-stage	T3	42 (66.7%)	21 (33.3%)	1.47	0.226
	T4	170 (58.4%)	121 (41.6%)		
N-stage	N0-1	120 (68.2%)	56 (31.8%)	10.02	0.002
	N2-3	92 (51.7%)	86 (48.3%)		
Overall stage	III	38 (66.7%)	19 (33.3%)	1.30	0.254
	IV	174 (58.6%)	123 (41.4%)		
Chemotherapy	Yes	197 (59.0%)	137 (41.0%)	2.02	0.156
	No	15 (75.0%)	5 (25.0%)		
GTVnx V95%	<95%	28 (37.3%)	47 (62.7%)	20.15	<0.001
	≥95%	184 (65.9%)	95 (34.1%)		
Prescribed dose	≤73.92 Gy	201 (63.0%)	118 (37.0%)	13.09	<0.001
	>73.92 Gy	11 (31.4%)	24 (68.6%)		

*Percentage = the number before the bracket divided by the numbers of before the bracket in the same line of column 3 plus column 4.

GTVnx: Primary gross target volume; GTVnx V95%: percentage volume of GTVnx receiving 95% prescribed doses.

### MRI imaging

MR imaging was performed with a 1.5-T unit— Siemens Vision Plus (Erlangen, Germany). The protocol was used for the NPC study including axial T1-weighted images without fat saturation, axial T2-weighted images, axial proton density images, sagittal T1-weighted images, and postcontrast axial, coronal and sagittal T1-weighted images with fat saturation. The upper extent covers 2 cm above the sella turcica and the lower extent reaches 2 cm below the lower edge of the clavicle. Axial T1-weighted fast spin-echo (FSE) images were obtained with repetition time msec/echo time msec of 600/15, echo train length of eight, two signals acquired, 24-mm field of view, 256 × 256 matrix, 5-mm-thick section, and 0.5-mm gap. Axial T2-weighted FSE images were obtained with 4200/102, echo train length of 16, two signals acquired, 20-mm field of view, 256× 256 matrix, 4-mm-thick section, and 0.4-mm gap. An intravenous bolus injection of 0.1 mmol/kg of body weight gadopentetate imeglumine (Magnevist; Schering, Berlin, Germany) was administered at 2 mL/sec for the contrast-enhanced series.

### Imaging evaluation

To reduce subjectivity, all patients were restaged according to the 7th edition of the American Joint Committee on Cancer (AJCC) Staging System for NPC; the pre- and post-treatment MRI scans for each patient were independently reviewed by two senior clinicians from the Departments of Radiology and Oncology. Local radiographic residual tumors were diagnosed by the consensus agreement of two head and neck radiologists and two senior radiation oncologists, respectively. Diagnostic criteria of residual tumors on MRI at different sites as follows: residual tumors present in the nasopharynx or other soft tissues following radiotherapy usually appeared as hypo-intense signal on T1-weighted imaging, as hyper-intensity signal on T2-weighted imaging and also exhibited enhancement following administration of Gd-DTPA. Regional lymph nodes were considered to have residual tumors on MRI if they were larger than 10 mm in short-axis diameter for cervical lymph nodes and larger than 5 mm for the retropharyngeal nodes at the end of radiotherapy. For the residual tumors at the skull base on MRI at the end of radiotherapy, we used the reference from previous reports [10,11]. It would be considered as residual tumors if the bone of the skull base was destructed with soft tissues and the degree and scope of strengthening of bone have not decrease compared to that of prior to chemoradiotherapy.

### Treatment

All patients underwent IMRT. The target volumes were defined with reference to International Commission on Radiation Units and Measurements (ICRU) reports No. 50 and No. 62. The primary tumor (GTVnx) and positive lymph nodes (GTVnd) were defined and the retropharyngeal lymph nodes were included in the GTVnx. The primary tumor before chemotherapy was delineated as the GTVnx for patients receiving neoadjuvant chemotherapy; two clinical target volumes (CTVs) were defined as follows: CTV1, the high-risk areas including a 5–10 mm extension around the GTVnx and other high-risk regions such as parapharyngeal space, inferior part of sphenoid sinus, posterior 1/3 of nasal cavity, posterior 1/3 of maxillary sinus, skull base, clivus, oval foramen, lacerated foramen and high-risk lymphatic drainage areas such as retropharyngeal lymph nodes, upper cervical lymph nodes levels II, III, and Va, 60 Gy irradiation was given; and CTV2, the low-risk lymphatic drainage areas including lower cervical lymph nodes levels IV and Vb, 50 Gy irradiation was given. The corresponding planning target volumes (PTVs) were generated by extending each CTV by 3 mm; the prescribed doses for the PGTVnx (GTVnx + 3 mm margin) were 66.0–75.9 Gy; GTVnd, 69.96–72.6 Gy; PTV1, 59.4–64.0 Gy, PTV2, 50.0–54.0 Gy. The doses to the PTV2 were administered over 28 fractions and other doses over 33 fractions; all patients were treated with simultaneous modulated accelerated radiotherapy once a day for five days a week. Dose limits for the critical tissue structures and plan evaluation were as defined by the Radiation Therapy Oncology Group (RTOG) 0225 [12]. The patients were re-examined by MRI when they finished radiotherapy or the radiation dose reached to approximately 70 Gy. The patient with significant residual tumors at the end of radiotherapy was observed or treated with an IMRT boost dose of 4–10 Gy to the residual lesions over 2–5 fractions depending on the individual toxicity (Organs at risk) and tumor response to radiotherapy. Chemotherapy was part of the treatment plan for all patients; 21 patients who were unwilling to receive chemotherapy or could not tolerate chemotherapy did not undergo chemotherapy. Neoadjuvant chemotherapy was administered when the waiting time for radiotherapy was longer than acceptable or to downsize bulky tumors. At the end of radiotherapy, adjuvant chemotherapy was administered to the patients with N2/N3 stage disease and with existing residual disease detected by MRI or physical examination.

### Follow-up

The follow-up methods included direct telephone calls to the patients or their families; or hospital visits for the patients. Follow-up was measured from the first day of treatment to last follow-up date or date of patient’s death. After radiotherapy, follow-up examinations were conducted once every 3 months in the first 2 years, once every 6 months in years 2 to 5, and annually thereafter. MRI of the nasopharynx and neck region was performed once a year for the patient with no residual tumor, or every 3–6 months for the patient with residual tumor.

Recurrence was defined as the tumor regrown after disappearing at least one month. The duration of overall survival (OS) was calculated from the day of radiotherapy completion to the date of patient’s death or last follow-up. The duration of local relapse-free survival (LRFS) was calculated from the day of radiotherapy completion to the date of tumor local recurrence. The duration of disease-free survival (DFS) was calculated from the day radiotherapy completion to the date of tumor recurrence, distant metastasis or death.

### Statistical analysis

All statistical analyses were performed using Statistical Package for the Social Sciences version 17.0 (SPSS, Chicago, IL, USA). Actuarial rates were calculated using the Kaplan-Meier method and differences were compared using the log-rank test. Multivariate analysis with the Cox proportional hazards model was used to test for independent significance by backward elimination of insignificant explanatory variables. The Mann–Whitney test was used to examine the between-group differences in the GTVnx V95% value. The criterion for statistical significance was set at α = 0.05 and *P*-values were based on two-sided tests.

## Results

### Clinical characteristics of the patients

The clinical characteristics of the patients are presented in Table 1. At the end of radiotherapy, 142/354 cases (40.1%) had residual tumors. The residual tumor rate for patients with N2-3 disease was significantly higher than that of patients with N0-1 disease (*P* = 0.002). The patients in the residual tumor group had a higher prescribed radiation dose than the patient in no residual tumor group (*P* < 0.001). The residual tumor rate was significantly lower in the patients with the minimum absorbed dose of the 95% GTVnx (GTVnx D95%) ≥ 70 Gy or GTVnx V95% ≥ 95% compared to the patients with a GTVnx D95% < 70 Gy (*P* < 0.001) or GTVnx V95% < 95% (*P* < 0.001), respectively. The locations and numbers of residual tumors are summarized in Table 2. As mentioned previously, only 11.44% of residual tumors were located in the pharyngonasal cavity, 30.93% in the skull base, and 30.15% in the parapharynx and other soft tissues, and 45.07% of patients had more than one residual tumor.

**Table 2:** **Characteristics of the 236 residual tumors from 142 patients with PNC at the end of IMRT**

**Characteristic**	**Number**	**Percentage (%)**
Residual tumor location		
Pharyngonasal cavity	27	11.44
Skull base	73	30.93
Parapharynx and Other soft tissues	72	30.15
Paranasal sinus	23	9.75
Intracranial space	9	3.81
Cervical lymph nodes	32	13.56
Total	236	100.00
Number of residual tumors*		
One tumor	78	54.93
More than one tumor	64	45.07
Total	142	100.00

Number of residual tumors* refers to the number of anatomic sites containing residual tumors including the pharyngonasal cavity, skull base, parapharynx and other soft tissues, intracranial space, and cervical lymph nodes. Some patients had more than one residual tumor.

### Treatment outcomes

The median follow-up period for all patients was 45 months (range, 3–78 months). In total, 22/346 cases developed local recurrence (6.36%), 55/346 developed distant metastasis (15.9%), and 9/346 developed recurrence plus distant metastasis (2.6%). Among the local relapse cases, 4 nasopharyngeal, 5 cervical lymph nodes, 6 bone of the skull base, 1 orbit, 2 paranasal sinus, 2 pharyngeal lymph nodes and 2 parapharyngeal spacerecurrences. Among the distant metastasis cases, 15 liver metastasis, 24 pulmonary metastasis, 20 bone metastasis, 1 adrenal metastasis, 1 retroperitoneal metastasis, and 10 multiple organs metastasis. There were 64 deaths out of 346 patients (18.5%) which 49 patients were due to tumor recurrence and metastasis, 10 were due to tumor-associated complications (5 cases nasopharyngeal hemorrhage, 1 case septic shock, 3 cases malnutrition systemic failure, and 1 case syncope), 1 patient was due to gastrointestinal bleeding and 4 patients were due to unknown causes.

### Prognostic value of residual tumors after IMRT

**Figure 1:**
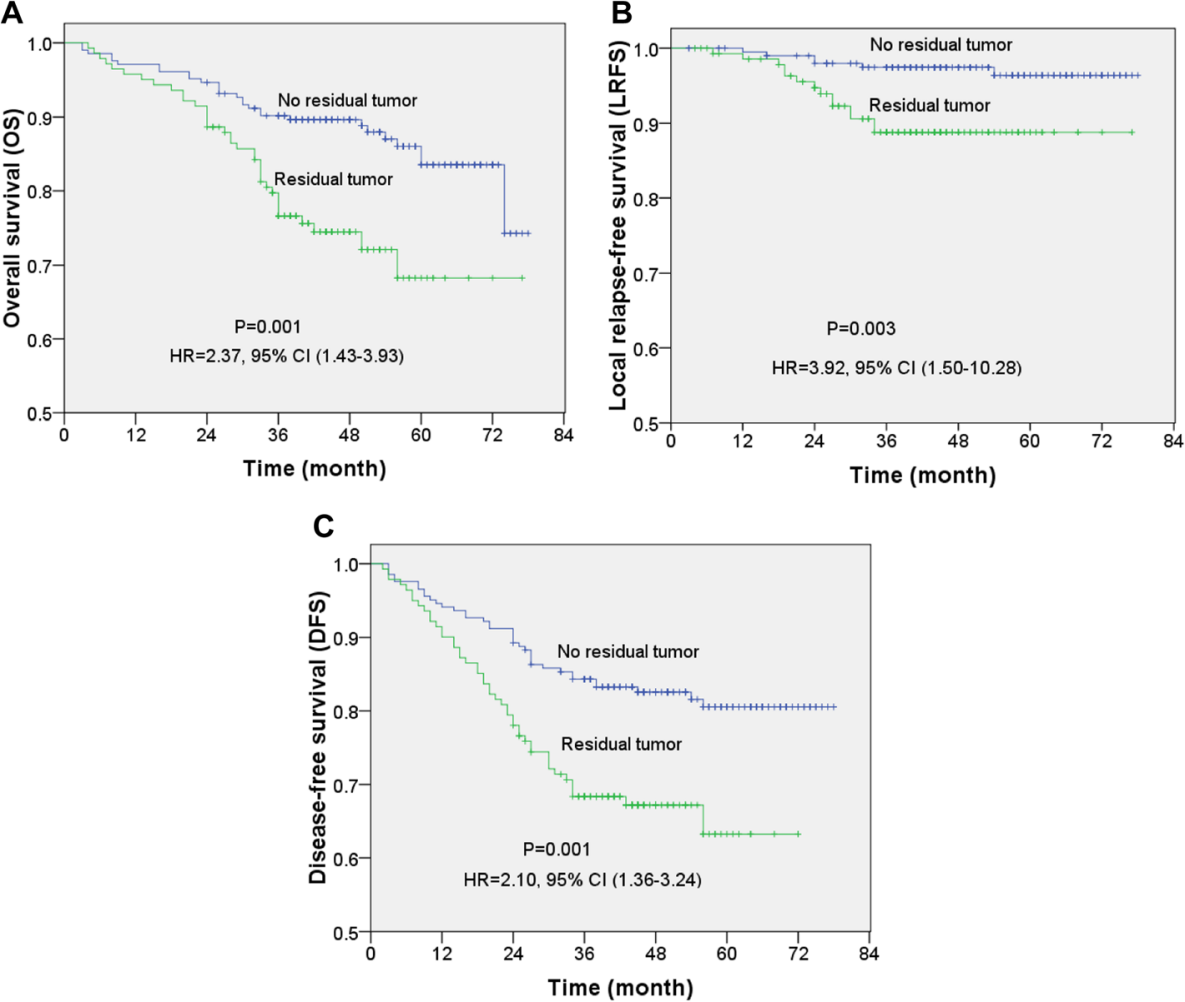
Kaplan-Meir survival curves for 346 patients with locally-advanced nasopharyngeal carcinoma (NPC). **(A)** Overall survival (OS), **(B)** local relapse–free survival (LRFS), **(C)** disease-free survival (DFS) for patients stratified by the presence and absence of a residual tumor at the end of IMRT. *P*-values were calculated using the unadjusted log-rank test; Hazard ratios (HR) were calculated using the unadjusted Cox proportional hazards model; 95% CI: 95% confidence interval.

Survival rates were calculated using the Kaplan–Meier method and compared using the log-rank test. The 3-year OS rate for the entire cohort study was 83%, 73% in the residual tumor group and 90% in the no residual tumor group (HR 2.15, 95% CI 1.21-3.82, *P* = 0.007); 3-year LRFS for the entire cohort study was 94%, 89% in the residual tumor group and 97% in the no residual tumor group (HR 4.46, 95% CI 1.61-12.38, *P* = 0.002); 3-year DFS for the entire cohort study was 76%, 67% in the residual tumor group and 82% in the no residual tumor group (HR 2.21, 95% CI 1.40-3.48, *P* = 0.001). Survival curves demonstrated that the presence of a residual tumor was associated with poorer survival outcomes, as shown in Figure 1, the statistically significant differences of the OS, LRFS and DFS survival curves between the residual tumor group and no residual tumor group (OS: HR = 2.37, 95% CI 1.43-3.93, *P* = 0.001; LRFS: HR = 3.92, 95% CI 1.50-10.28, *P* = 0.003; DFS: HR = 2.10, 95% CI 1.36-3.24, *P* = 0.001).

The Univariate analysis suggests that the factors influencing the 3-year OS rate are age (P = 0.002), N-stage (P = 0.003), overall stage (P = 0.038), and presence or absence of a residual tumor at the end of radiotherapy (P = 0.001), the factors influencing LRFS are age (P = 0.001) and presence or absence of a residual tumor at the end of radiotherapy (P = 0.003), and the factors influencing DFS are age (P = 0.008), N-stage (P = 0.024), T-stage (P = 0.05), overall stage (P = 0.024), and presence or absence of a residual tumor at the end of radiotherapy (P = 0.001). However, chemotherapy and prescribed radiation dose are not the factors for significantly influencing the OS, LRFS, or DFS (Table 3).

**Table 3:** **Univariate analysis of prognostic factors in patients with NPC**

**Variable**	**No.** ^**#**^ ** N = 346**	**3-year OS (%)**	**p-value**	**3-year LRFS (%)**	**p-value**	**3-year DFS (%)**	**p-value**
Age			0.002		0.001		0.008
<50 y	225	87		97		80	
≥50 y	121	75		87		69	
N-stage			0.003		0.685		0.024
N0	63	92		94		84	
N1	110	87		92		77	
N2	113	84		94		77	
N3	60	67		96		65	
T-stage			0.105		0.838		0.050
T3	59	90		95		88	
T4	287	82		93		74	
Overall stage			0.038		0.684		0.024
III	53	92		94		91	
IV	293	82		94		74	
Chemotherapy			0.386		0.059		0.427
Yes	327	84		94		77	
No	19	72		83		68	
Prescribed dose			0.926		0.985		0.793
≤73.92 Gy	311	83		93		76	
>73.92 Gy	35	85		94		75	
Residual tumor*			0.007		0.002		<0.001
No	205	90		97		82	
Yes	141	73		89		67	

*Residual tumor: tumor detected by MRI at the end of radiotherapy.

^#^No.: number of patients.

OS: overall survival; LRFS: local relapse-free survival; DFS: disease-free survival.

The following factors were associated with treatment outcomes in multivariate analysis (Table 4): age (HR 2.32, *P* = 0.001), N-stage (HR 1.43, *P* = 0.008), and the presence or absence of a residual tumor after radiotherapy (HR 2.11, *P* = 0.004) were significantly associated with OS; age (HR 4.80, *P* = 0.002), and the presence or absence of a residual tumor after radiotherapy (HR 4.80, *P* = 0.002) were significantly associated with LRFS; age (HR 1.81, *P* = 0.007), N-stage (HR 1.26, *P* = 0.049), and the presence or absence of a residual tumor after radiotherapy (HR 1.91, *P* = 0.004) were significantly associated with DFS.

**Table 4:** **Summary of multivariate analysis of prognostic factors in patients with NPC**

**End point**	**Variable**	**Regression coefficient**	**Standard error**	**HR**	**95% CI**		***P*** **-value**
					**Lower**	**Upper**	
OS	Age	0.84	0.25	2.32	1.412	3.79	0.001
	N-stage	0.36	0.14	1.43	1.10	1.87	0.008
	Residual tumor	0.75	0.26	2.11	1.26	3.54	0.004
LRFS	Age	1.57	0.50	4.80	1.78	12.83	0.002
	Residual tumor	1.59	0.51	4.88	1.81	13.16	0.002
DFS	Age	0.59	0.22	1.81	1.17	2.80	0.007
	N-stage	0.23	0.12	1.26	1.01	1.58	0.049
	Residual tumor	0.65	0.23	1.91	1.23	2.98	0.004

HR: hazard ratio; CI, confidence interval; *P*-values were calculated using an adjusted Cox proportional hazards model. OS: overall survival; LRFS: local relapse-free survival; DFS: disease-free survival.

The variables were stratified as following: Age: < 50 y vs ≥ 50 y; N-stage: N0 vs N1vs N2 vs N3; T-stage: T3 vs T4; Residual tumor: having tumor vs no tumor. The table presents only the data with statistical significances.

### Relationship between the radiation dose and the presence of residual tumors at the end of IMRT

Prescribed radiation doses for all patients in the study were ranged from 62.72 Gy to 80.64 Gy with a mid-value of 73.92 Gy. A higher prescribed dose (>73.92 Gy) was only given to the patients who still had a large residual tumor and the OARs (Organs at risk) were not at risk to accept radiation boost. However, the data show that increase of radiation doses did not reduce the rate of residual tumor, the residual rates were 68.6% and 37.0% for the groups of high prescribed dose and lower prescribed dose, respectively; or did not improve the treatment outcomes (Table 5). The 3-year OS, LRFS, and DFS rates were 83% vs 85%, 93% vs 94% and 76% vs 75% for the groups of high prescribed dose and low prescribed dose, respectively (Table 3). There was not statistically significantly different (p > 0.05). The median GTVnx V95% values for the higher radiation group and the low dose group were not significantly different (99.1% vs. 98.7%, *P* = 0.583), indicating that the GTVnx V95% cannot be improved by increasing the radiation dose. However, the median GTVnx V95% values were significantly different (97.2% vs 99.5%, *P* < 0.001) between the residual tumor group and no residual tumor group, indicating that a low GTVnx V95% may be associated with the presence of a residual tumor after treatment (Table 5).

**Table 5:** **Relationship of PGTVnx V95% and prescribed radiation dose and residual tumor after IMRT**

**Variable**	**Group**	**GTVnx V95%**				
		**Median**	**Minimum**	**Maximum**	**Interquartile range**	***P*** **-value**
Prescribed dose	>73.92 Gy	99.1	89.9	100	2.9	0.583
	≤73.92 Gy	98.7	84.4	100	4.0	
Residual tumor	Yes	97.2	84.4	100	5.1	0.001
	No	99.5	85.0	100	2.5	

GTVnx V95% = the percentage volume of the GTVnx receiving 95% of the prescribed dose.

## Discussion

The present study reveals that advanced N-stage, a low GTVnx V95% were associated with a higher risk of a residual tumor in the patients with locally-advanced NPC. The presence of a residual tumor at the end of IMRT was a significant independent factor for OS, LRFS and DFS in the patients with locally-advanced NPC. Additionally, a higher prescribed radiation dose was not associated with a high GTVnx V95% and did not improve survival outcome.

In this cohort study of 358 patients with NPC, MRI indicated that 142 patients had residual tumors in total of 236 (some patients had more than one tumor) at the end of IMRT. only 27 tumors were located in the nasopharynx while most tumors were located in the skull base, soft tissues or other tissues that cannot be assessed by biopsy (Table 2). Chan et al. [13] reported that deeply seated residual/recurrent tumors beyond the reach of routine nasopharyngeal biopsy are not rare (15.4%). However, only less than half of the patients had locally advanced diseases in their study. To understand the status of residual tumors at the end of radiation, only imaging tools CT/MRI/PET are suitable to be used. MRI has become the gold standard for NPC diagnosis and therapeutic evaluation due to the high accuracy and economic cost-effectiveness. The specificity of MRI was reported at the range of 44–83% [14-16]. Post-irradiation inflammatory changes, such as an immature scar, reactive mucosal and submucosal changes or osteoradionecrosis, may interfere with the interpretation of MRI and thus decrease its specificity. In contrast, 18F-FDG PET showed a significantly higher specificity of 93.4% in the assessment of treatment response and appears less influenced by radiotherapy (RT)-induced inflammation [14]. A systematic review suggests that FDG-PET is the best modality for diagnosis of local residual or recurrent NPC [16]. However, the results may be disputed by other report showing that there was a trend toward greater overall accuracy of MRI over PET/CT in detecting residual and/or recurrent NPC at the primary site; 92.1% for MRI and 85.7% for FDG PET/CT (P = 0.16) [9]. Because the intracranial localizations and the perineural spreads via the foramen ovale, the anterior foramen lacerum, or the pterygopalatine fossa were depicted with MRI only, FDG PET/CT is not considered as a good method for the study of the intracranial disease due to the physiologically high FDG uptake by the brain.

In the present study,the MRI-derived residual tumor rate was 40.1% (142/358), similar to the study of Han et al. [17] showed the residual tumor rates of 44.2% (72/196) in the patients treated with IMRT and 26.6% (52/196) in the patients treated with conventional radiotherapy (CRT) detected by nasopharyngeal MRI at the end of radiotherapy. Zhang et al. [18] reported that it has been achieved 40.4% complete response of primary tumor (CRPT), 44.7% partial response of primary tumor (PRPT), and 14.9% stable disease of primary tumor (SDPT) at the end of radiotherapy in a study with 188 NPC patients. Lin et al. [19] reported that 50% (54/108) of residual tumors were detected by MRI in the patients with NPC one month after completing radiotherapy. However, Chan et al. reported only 3.6% (4/112) of the patients were found to have residual tumors 3 months after radiotherapy by MRI or PET-CT [13]. The reason for greatly different rates of residual tumors reported above may be due to multiple factors including differences in tumor staging, tumor sites, the use of different diagnostic criteria for MRI, technologies of radiotherapy [IMRT or CRT or intracavitary brachytherapy (ICBT)] and the application of comprehensive treatments and so on. In our study, the results in Table 1 suggest that the presence of residual tumors is associated with the latter N stage and the GTVnx V95%.

Another important factor is the time point for evaluation of the residual tumors. Tumor response to chemoradiotherapy is time-dependent so a positive histological result after radiotherapy may become negative after 12 weeks [20]. This is why the residual tumor rate is so low in the study of Chan et al. and most oncologists prefer to assess the residual tumor at the time of three months after radiotherapy. However, it may reduce the efficacy of chemoradiotherapy at three months after radiotherapy due to delayed treatment for the residual tumor. Early intervention to modify the treatment strategy may improve the treatment outcome for patients with residual tumors. It is still controversial whether or not the tumor regression rate during or after treatment is correlated with the survival rate in the patients with NPC. Wang et al. [21] reported that the patients who achieved a slow response (CR when the radiation dose was around 70 Gy) had a significantly better prognosis than the patients who achieved rapid regression (CR when the radiation dose was within 50 Gy). Moreover, the prognosis of both of CR groups was significantly better than the patients with a residual tumor at the end of radiotherapy. However, this study was based on the patients who received conventional radiotherapy. Recently, Zhang et al. [18] found that the 5-year OS rates for the patients with CRPT, PRPT, and SDPT at the end of radiotherapy were 84.0%, 70.7%, and 44.3%, respectively (HR = 2.177, P < 0.001). However, approximately one third of the patients had stage T1 or T2 disease and 55.9% of the patients were treated with conventional 2-dimensional radiotherapy (2D-CRT) in their study. The radiobiological effect of IMRT is different from conventional radiotherapy, residual lesions after IMRT have always been received a radical radiation dose. Dose the residual lesions still have an impact on the survival of the patients with NPC? In our study, all patients undergoing IMRT and residual tumors at the end of radiotherapy are still an important negative prognostic factor for OS (3 year OS was 73% vs 90% in the residual tumor group and in the no residual tumor group, *P* = 0.007), LRFS (3-year LRFS was 89% vs 97% in the residual tumor group and in the no residual tumor group, P = 0.002), and DFS (3-year DFS was 67% vs 82% in the residual tumor group and in the no residual tumor group, *P* < 0.001). However, Fang et al. [22] reported that the regression rates for the primary tumors and lymph nodes were not significantly associated with local or regional recurrence and overall survival with radiotherapy at 45 Gy. Mantyla et al. [1] reported that in patients with early-stage head and neck cancer (T1-2N0), a significantly more favorable prognosis was observed if the tumor was disappeared by the mid-point of treatment (30 Gy) than that of tumor disappeared by the end of treatment. On the contrary, the prognosis for patients with advanced disease was significantly more favorable if the tumor was disappeared at the end of radiotherapy (2D-CRT) than that of tumors disappeared by the mid-point of treatment. The longer the time after radiotherapy, the more the tumor will regress. Therefore, it needs more times for tumor regression in locally-advanced tumor than in early-stage tumor. It suggests that it is not a good practice to evaluate curative effect too early for the patients with locally advanced NPC.

The prognosis of patients with NPC is related to numerous clinical factors such as age, clinical stage, chemotherapy, radiation dose, and so on. In our study, age is an independent prognostic factors for OS (HR = 2.32, P = 0.001), LRFS (HR = 4.80, P = 0.002), and DFS (HR = 1.81, P = 0.007). It is consistent with the results reported by Erkal et al. [23]. N stage is also an independent prognostic factor for OS (HR = 1.43, P = 0.008) and DFS (HR = 1.26, P = 0.049). Clinic staging had a very certain influence on the prognosis of NPC [24]. However, the influence of T stage on the prognosis of NPC becomes smaller with the application of IMRT. In one of the recent studies, multivariate analyses of 305 patients undergoing IMRT revealed that T-classification had no predictive value for local control and survival, whereas only N-classification was a significant prognostic factor for OS [25]. In our study, T-stage also had no predictive value for OS, and LRFS (Table 3). The 7th edition of the AJCC Staging System was adopted in our study and medial or lateral pterygoid involvement was staged as T4. However, medial and/or lateral pterygoid involvement was staged as T3 or T2 by other studies due to better prognosis compared to skull base involvement which being staged as T3 [26,27]. The reason of T stage and overall stage has no significant effect on NPC may be caused by the pitfall of the 7th AJCC Staging System. The effect of adjuvant chemotherapy on prognosis has been disputed for a long time, study showed that patients treated with chemotherapy as an adjuvant to radiotherapy had a better DFS compared to the patients without adjuvant chemotherapy (P = 0.04) [28]. Univariate analysis in our study also showed that the rates of 3 year OS (84% vs 72%), LRFS (94% vs 83%), and DFS (77% vs 68%) were slight improved in the chemotherapy group compared to the group without chemotherapy, but the difference was not statistically significant (P > 0.05). The results may be affected due to too small numbers with the group without chemotherapy (n = 19).

In the present study, prescribed higher radiation doses did not improved the survival outcome by both univariate and multivariate analyses. It has reported that IMRT has improved the treatment outcome in the patients with NPC [29]. IMRT offers a number of advantages over conventional radiotherapy in the terms of target conformity and the ability to increase the radiation dose to the target volume while sparing the surrounding normal organs at risk [30]. Study from a Hong Kong group found that it was a significant determinant of progression-free survival and distant metastasis-free survival for advanced T-stage tumors when the doses were escalated to above 66 Gy in IMRT-based therapy [31]. However, despite the advancement in IMRT dosimetric inadequacy remains a significant problem when a tumor invades directly into critical neurological OARs such as optic chiasm, brainstem and spinal cord [32]. In the present study, most patients who had residual tumors received a radical radiation dose: 67.6% patients (96/142) received a GTVnx V95% ≥ 95% and 83.1% patients (118/142) received a D95% ≥ 66 Gy. From the data in Table 5, we can see that the median GTVnx V95% value for the patients in the residual tumor group (97.2%) was significantly lower than that of the patients in the no residual tumor group (99.5%; *P* < 0.001). This indicates that the GTVnx V95% is one factor associated with the presence of residual tumors after treatment. However, the median GTVnx V95% values were not significantly different (P > 0.05) between the patients in the group of prescribed high doses > 73.92 Gy and in the group of low doses ≤ 73.92 Gy. The data indicate that increase of the prescribed dose cannot improve the GTVnx V95% and it may due to that the radiation dose in the tumor near the OAR had not been improved, instead of increased radiation dose in the normal tissues to lead to an increased risk of radiotherapy complications. There were 10 deaths from radiotherapy complications in our study. This may be the reason that elevation of prescribed radiation doses cannot improve survival outcome.

In the present study, we demonstrate that the presence of a MRI-derived residual tumor after IMRT is an important negative prognostic factor in the patients with locally-advanced NPC. However, this study has several limitations. First, this investigation was a retrospective analysis. Second, the follow-up time (3–6 years) may be too short to detect a relapse of tumor. Moreover, MRI has some shortcoming, we cannot rule out the possibility that abnormal signals on MRI may be due to a reactive change as a result of radiotherapy without the verification by pathology or PET-CT.

## Conclusions

The presence of a residual tumor detected by MRI at the end of IMRT in patients with locally-advanced NPC is closely associated with a poor prognosis, which suggests a potential role for MRI in predicting local control and prognosis in patients with NPC. Additionally, elevating the prescribed dose after radical radiotherapy did not increase the GTVnx V95% or improve the outcome of survival in locally-advanced NPC received IMRT treatment.
